# Iron chelation and 2‐oxoglutarate‐dependent dioxygenase inhibition suppress mantle cell lymphoma's cyclin D1

**DOI:** 10.1111/jcmm.14655

**Published:** 2019-09-13

**Authors:** Olga Babosova, Katarina Kapralova, Leona Raskova Kafkova, Vladimir Korinek, Vladimir Divoky, Josef T. Prchal, Lucie Lanikova

**Affiliations:** ^1^ Department of Cell and Developmental Biology Institute of Molecular Genetics Academy of Sciences of the Czech Republic Prague Czech Republic; ^2^ Department of Biology Faculty of Medicine and Dentistry Palacky University Olomouc Olomouc Czech Republic; ^3^ Division of Hematology & Hematologic Malignancies Department of Internal Medicine University of Utah School of Medicine and VAH Salt Lake City Utah

**Keywords:** 2‐oxoglutarate‐dependent enzymes, cell cycle, DNA damage, iron, mantle cell lymphoma, prolyl hydroxylases (*EGLN*/PHDs)

## Abstract

The patients with mantle cell lymphoma (MCL) have translocation t(11;14) associated with cyclin D1 overexpression. We observed that iron (an essential cofactor of dioxygenases including prolyl hydroxylases [PHDs]) depletion by deferoxamine blocked MCL cells’ proliferation, increased expression of DNA damage marker γH2AX, induced cell cycle arrest and decreased cyclin D1 level. Treatment of MCL cell lines with dimethyloxalylglycine, which blocks dioxygenases involving PHDs by competing with their substrate 2‐oxoglutarate, leads to their decreased proliferation and the decrease of cyclin D1 level. We then postulated that loss of *EGLN2*/PHD1 in MCL cells may lead to down‐regulation of cyclin D1 by blocking the degradation of FOXO3A, a cyclin D1 suppressor. However, the CRISPR/Cas9‐based loss‐of‐function of *EGLN2*/PHD1 did not affect cyclin D1 expression and the loss of FOXO3A did not restore cyclin D1 levels after iron chelation. These data suggest that expression of cyclin D1 in MCL is not controlled by *ENGL2*/PHD1‐FOXO3A pathway and that chelation‐ and 2‐oxoglutarate competition‐mediated down‐regulation of cyclin D1 in MCL cells is driven by yet unknown mechanism involving iron‐ and 2‐oxoglutarate‐dependent dioxygenases other than PHD1. These data support further exploration of the use of iron chelation and 2‐oxoglutarate‐dependent dioxygenase inhibitors as a novel therapy of MCL.

## 1 INTRODUCTION

Mantle cell lymphoma (MCL) is an incurable B‐cell lymphoma characterized by a translocation that juxtaposes the *CCND1* gene (which encodes *cyclin D1*, *CD1*) on chromosome 11q13 and an immunoglobulin heavy chain gene promoter on chromosome 14q32. MCL represents a small portion of malignant lymphomas with historically poor long‐term survival.^1^ The combination of intensive chemo‐immunotherapy containing rituximab and cytarabine, with or without autologous stem cell transplantation consolidation, led to substantial improvements in patient outcome in recent years, but none of them is curative.^1^, ^2^ Novel therapeutic approaches incorporate lenalidomide (an immunomodulatory agent), bortezomib (a proteasome inhibitor) and ibrutinib and acalabrutinib (both Bruton kinase inhibitors), when especially ibrutinib has impressive responses, but with almost uniform development of resistance.^1^, ^2^, ^3^ Developing precise therapeutic strategy that will prevent the relapses and allow long‐term remission without excess toxicities is still a serious challenge. We observed that MCL‐derived cell lines have decreased survival and proliferation compared to non‐MCL lymphoma cell lines when grown at iron‐deprived conditions,^4^ and Vazana‐Barad et al^5^ reported that MCL patients may benefit from iron‐chelating agents.

Iron is essential for cell proliferation. In tumour cells, the iron metabolic alterations (an elevated entry, a decrease in its elimination and a disruption of its storage) help to facilitate the accelerating cell division and supply iron for increased DNA synthesis but, on the other hand, also sensitizes cancer cells to iron depletion.^6^, ^7^ Although iron chelators were developed for treatment of iron overload diseases, they are also potent DNA synthesis inhibitors in vitro, as they inhibit ribonucleotide reductase (RR),^8^, ^9^ an iron‐dependent enzyme, essential for reduction of ribonucleotides to deoxynucleotides (dNTPs).^9^, ^10^, ^11^ Iron chelators therefore represent promising adjuvant elements in the treatment of cancers especially in subgroup of patients dealing with resistance to the established therapy.

It has been shown that in MCL cell lines, iron chelator deferasirox down‐regulates cyclin D1 which in turn leads to inhibition of Rb phosphorylation and increase of the E2F/Rb complex levels ultimately leading to G1/S arrest.^5^ The mechanism by which iron may affect cyclin D1 in cancer cells has been suggested by Nurtjahja‐Tjendraputra et al^12^ in a study that examined the ability of iron chelators to inhibit cell proliferation and induce apoptosis. It was postulated that iron chelation caused proteasomal degradation of cyclin D1. The degradation of cyclin D1 was ubiquitin independent in iron‐deplete conditions, while ubiquitination of cyclin D1 degradation takes place in iron‐replete cells.

However, Zhang et al^13^ showed that the mammalian cyclin D1–dependent proliferation is regulated by prolyl hydroxylase 1 (PHD1, encoded by the *EGLN2* gene) in a hypoxia‐inducible factor independent manner by the transcriptional mechanism rather than via the proteasomal pathway. Cyclin D1 is not a direct substrate for PHD1. It was suggested that forkhead box O3A (FOXO3A) transcription factor is the link between the regulation of cyclin D1 and prolyl hydroxylase PHD1.^14^ PHD1 can hydroxylate FOXO3A on two specific prolyl residues thereby blocking its interaction with the USP9x deubiquitinase and promoting its proteasomal degradation. Loss of *EGLN2*/PHD1 leads to accumulation of FOXO3A, which, in turn, suppresses cyclin D1 expression.

Prolyl hydroxylases (PHDs) belong to the iron and 2‐oxoglutarate (2‐OG)–dependent dioxygenase family, and as principal negative regulators of HIFs, they contribute to oxygen sensing. There are three paralogues of the *EGLN* gene family (*EGLN1*/PHD2, *EGLN2*/PHD1 and *EGLN3*/PHD3) encoding PHDs. The PHD1 is found to be exclusively present in the nucleus, and PHD2 is mainly located in the cytoplasm while PHD3 protein is homogenously distributed throughout the cytoplasm and nucleus.^15^, ^16^ While all members of the PHD protein family contribute to regulation of cellular O_2_ sensing, only *EGLN2*/PHD1 and *EGLN3*/PHD3 were demonstrated to have HIF‐independent functions, such as in DNA damage control^17^, ^18^ and NF‐κB activity regulation.^19^, ^20^ The connection between prolyl hydroxylases and cell cycle regulation was first described in drosophila; their PHD homologue Hif‐1 prolyl hydroxylase (Hph) was shown to be a regulator of cellular growth and a key mediator for the drosophila cyclin–dependent protein kinase complex cyclin D/cyclin‐dependent kinase 4.^21^ The mouse PHD1 homologue *Falkor* was identified as a DNA damage–related growth regulator in mouse embryonic fibroblasts.^17^ It was shown that Falkor can also inhibit HIF‐2 and a combined knockout of *EGLN2* and *EGLN3* leads to polycythemia/erythrocytosis as HIF‐2 is the principal regulator of erythropoietin gene.^22^, ^23^ In human breast cancer cells, *EGLN2* mRNA was shown to accumulate in cells stimulated with oestrogen and participate in oestrogen‐independent cancer cells’ growth and their resistance to hormone therapy.^24^

In the present study, we confirmed the effect of cellular iron depletion on MCL cell lines^5^, ^12^ and observed increased sensitivity to chelation treatment of MCL cell lines in comparison with the non‐MCL cell lines without constitutively active cyclin D1. As the molecular mechanism inducing cyclin D1 degradation after iron chelation is not known, we postulated that it could be linked to PHD1‐FOXO3A pathway. To unravel the role of prolyl hydroxylases in cyclin D1 regulation in MCL, we generated MCL cell lines harbouring the *EGLN2* or *FOXO3A* loss‐of‐function (LOF) genes. In addition, MCL cells were treated with 2‐OG analogue, dimethyloxalylglycine (DMOG), a competitive inhibitor of prolyl hydroxylase domain‐containing proteins. Several PHD inhibitors have been recently generated by Pharma industry, and they are already used in clinical trials of anaemia^25^, ^26^, ^27^, ^28^; further, the inhibitors of PHDs that target HIF‐2α are already used in the clinical trials of HIF‐dependent cancers.^29^, ^30^ These inhibitors have different selectivity against 2‐OG‐dependent oxygenases,^31^, ^32^ but in addition to 2‐OG oxygenase inhibitory potency can exhibit also iron‐chelating ability.^31^ We propose that either chelating agents or broad spectrum 2‐OG‐dependent oxygenase inhibitors (rather than specific PHD inhibitors) can be expeditiously applied as a new avenue for MCL‐targeted therapy.

## 2 MATERIALS AND METHODS

### 2.1 Cell culture

Human MCL cell lines Jeko‐1 and Mino were a kind gift from Dr Jianguo Tao at the H. Lee Moffitt Cancer Center & Research Institute. The HBL‐2 cell line was a kind gift from Dr Elliot Epner at Oregon Health and Science University. We purchased SUDHL‐6 (CRL‐2959™), DG‐75 (CRL‐2625™) and HEK293 (CRL‐1573™) from ATCC. All cell lines were maintained in RPMI medium 1640 with GlutaMAX (ThermoFisher Scientific), supplemented with 10% foetal bovine serum (ThermoFisher Scientific), and treated with 100 U/mL penicillin and 100 μg/mL streptomycin (both ThermoFisher Scientific) in a humidified atmosphere containing 5% CO_2_ at 37°C. The treatments of the cells by deferoxamine mesylate salt (250 µmol/L, DFO, Sigma‐Aldrich) and dimethyloxalylglycine (1 mmol/L, DMOG, Sigma‐Aldrich) are indicated in the corresponding figures and legends. For hypoxia induction, cells were cultured 24 hours in hypoxia chamber (StemCell Technologies) containing certified gases mixture (1% O_2_, 5% CO_2_, 94% N_2_), which was placed in the standard tissue culture incubator at 37°C. Cultures and assays used for analyses of mouse embryonic stem cells (mESCs) are described in Appendix S1.

### 2.2 Proliferation assay

Cell number and viability were determined using CellometerAutoT4 (Nexcelom Bio‐science) based on the trypan blue exclusion method or by CellTitre‐Blue reagent (Promega) and Perkin‐Elmer Envision analyzer.

### 2.3 Cell cycle and apoptosis analysis

Cell cultures were synchronized by serum starvation as described elsewhere.^6^ Briefly, cells were washed with PBS and serum‐starved for 24 hours at 37°C. Starved cells were stimulated with 10% FBS for 16 hours at 37°C in the presence or absence of 250 μmol/L DFO. Cells were harvested and washed with ice‐cold PBS and fixed with 70% ethanol, and the cell cycle was analysed using a BD FACSCanto II flow cytometer (BD Biosciences) and FlowJo™ software. Apoptosis was evaluated by flow cytometry using an Annexin V‐FITC Kit apoptosis detection kit (Miltenyi Biotec). Data were acquired by at least 10 000 cells using BD FACSCanto II instrument.

### 2.4 Western blot analysis

Cells were harvested in RIPA buffer (Sigma‐Aldrich) supplemented with a cocktail of protease inhibitors. Proteins were resolved on SDS‐polyacrylamide gels and electro‐blotted onto PVDF membranes (Millipore) or nitrocellulose membranes (Biorad). Membranes were incubated with following rabbit anti‐human primary antibodies: cyclin D1 (#2922S; Cell Signaling, 1:1000, lot:3), actin (Sigma‐Aldrich, 1:1000), HSP90 (#4877; Cell Signaling, 1:2000), FOXO3A (#2497; Cell Signaling, 1:1000), PHD1 (NB100‐310; Novus Biologicals, 1:500), phospho‐histone H2AX (Ser139; #9718; Cell Signaling, 1:1000) and mouse anti‐human primary antibody CtBP (sc‐17759; Santa Cruz, 1:1000) at 4°C overnight, washed in PBS with 0.05% Tween 20, and incubated for 1 hour with goat anti‐rabbit or goat antimouse horseradish peroxidase (HRP)–conjugated secondary antibody (ThermoFisher Scientific). HRP activity was detected with an ECL detection kit (Pierce, ThermoFisher Scientific).

### 2.5 RNA isolation and quantitative RT‐PCR

RNA was isolated using TRI reagent (Sigma‐Aldrich), and 500 ng of DNA‐free RNA was reverse‐transcribed using the First Strand cDNA Transcriptor Synthesis Kit (Roche) or 1000 ng of DNA‐free RNA was reverse‐transcribed using the RevertAid Reverse Transcriptase (ThermoFisher Scientific) according to the manufacturer's manual. Gene expression experiments were performed on LightCycler 480 system (Roche) with following TaqMan probes: Hs00765553_m1 *CCND1*, Hs00153380_m1 *CCND2*, Hs00236949_m1 *CCND3*, Hs00254392_m1 *EGLN1*, Hs00363196_m1 *EGLN2*, Hs00222966_m *EGLN3*, Hs00900055_m1 *VEGFA*, Hs00892681_m1 *SLC2A1*, Hs00175976_m1 *HK1*, Hs00818121_m1 *FOXO3A* and reference genes 4333761F *RPLP0* and 4333767F *GUSB*. All experiments were investigated in triplicate. The data reported represent the mean of three independent experiments; T bars designate SD. For statistical analysis, Student's paired *t* test with unequal variance was employed and *P* values <.05 were considered statistically significant.

### 2.6 Plasmids, virus production and infection

Lentiviral LentiCas9‐Blast (Addgene plasmid #52962) and LentiGuide‐Puro (Addgene plasmid #52963) single guide RNA plasmids were processed according to Lentiviral CRISPR ToolBox protocol GeCKO. Briefly, 293T packaging cell line was used for LentiCas9 amplification using packaging plasmids pVSVg (Addgene #8454) and psPAX2 (Addgene #12260). The viruses were collected 24 hours after transfection, precipitated with PEG‐it reagent (System Biosciences), and Mino cells were infected in the presence of 4 µg/mL Polybrene (hexadimethrine bromide) prior to drug selection (blasticidin 18 µg/mL) to produce cell line stably expressing Cas9 (Mino LentiCas9). The LentiCRISPR single guide RNA plasmid was digested by *BsmBI* enzyme, purified from agarose gel (Roche, High Pure PCR Product Purification Kit) and ligated with phosphorylated and annealed oligo pairs for single guide RNA (target sequence for *FOXO3A* 5′ GTGGGTACGCACCTTCCAGC 3′, for *EGLN2/PHD1* 5′ *TGATGCAGCGCC CATCGCCG* 3′). Mino LentiCas9 cell line was infected as described above in the presence of 4 µg/mL Polybrene prior to drug selection (puromycin 1 µg/mL).

## 3 RESULTS

### 3.1 The effect of cellular iron depletion on human mantle cell and non‐mantle cell lymphoma cell lines

**Figure 1:**
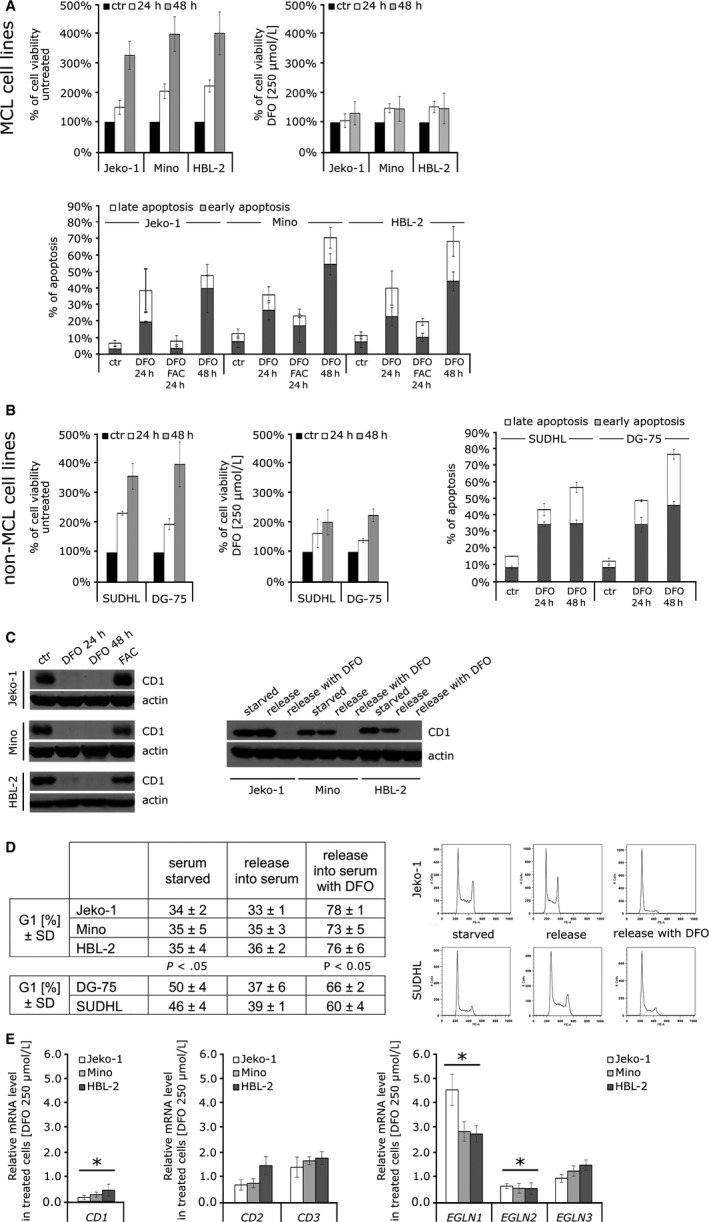
The effect of cellular iron depletion on human mantle cell lymphoma cell lines (Jeko‐1, Mino and HBL‐2) and two lymphoma cell lines (SUDHL‐6 and DG‐75) which do not harbour t(11;14)(q13;32) translocation. The data reported represent the mean of three independent experiments; T bars designate SD. For statistical analysis, Student's paired *t* test with unequal variance was employed and *P* values <.05 were considered statistically significant. A, (Upper panel) Proliferation rates during deferoxamine (DFO) (250 µmol/L) treatment in mantle cell lymphomamantle (MCL) cell lines. Iron chelator DFO inhibited the growth of all MCL cell lines. The results are demonstrated as percentage of cell growth in comparison with number of cells at time 0. (Lower panel) Percentage of total apoptotic cells (divided into early and late fractions) during DFO treatment. All cell lines exhibited increase in the percentage of apoptotic cells already after 24 h after iron depletion but co‐incubation of DFO‐treated cells with iron source FAC (100 µg/mL) abolished the effect. B, (Left panels) Proliferation rates during DFO treatment (250 µmol/L) in non‐MCL cell lines. The growth of non‐MCL cell lines was decreased but not fully inhibited. The results are demonstrated as percentage of cell growth in comparison with number of cells at time 0. (Right panel) Percentage of total apoptotic cells (divided into early and late fractions) during DFO treatment. Both lines exhibited increase in the percentage of apoptotic cells after 24‐h treatment. C, (Left panel) Treatment with DFO (250 µmol/L) depleted cyclin D1 protein level. The level of cyclin D1 was not detectable in MCL cell line treated with DFO already after 24 h. Re‐incubation of DFO‐treated cells with FAC (100 µg/mL) for 24 h restores cyclin D1 protein levels. SUDHL‐6 and DG‐75 cell lines have undetectable level of cyclin D1 (data not shown). (Right panel) Cyclin D1 protein level in MCL cell lines after serum starvation (24 h) and after the release into the medium with 10% FBS or 10% FBS with DFO (250 µmol/L). D, Cellular iron depletion sensitizing MCL cell lines to G1/S arrest. MCL cell lines do not stop cell cycle under serum‐starved condition and release with medium containing 10% FBS and DFO (250 µmol/L) sensitizing them to G1/S arrest (comparing to non‐MCL cell lines SUDHL‐6 and DG‐75). Data are demonstrated as percentage of cells in G1 phase. Representative cell cycle histograms analysis by FlowJo software. E, Expression analysis of selected genes in DFO‐treated (250 µmol/L) MCL cell lines after 24 h was determined by quantitative PCR. (Left panel) Treatment with DFO significantly decreases mRNA expression of *cyclin D1*. (Middle panel) The mRNA expression of *cyclin D1* homologs, *cyclin D2 *(*CD2*) and *cyclin D3 *(*CD3*) is not significantly affected by DFO treatment. (Right panels) Expression of *EGLN1* gene is significantly increased, the expression of *EGLN2* is significantly decreased, and the expression of *EGLN3* is not affected by DFO treatment

We extended our previous experiments^4^ and confirmed that iron chelation inhibits cell growth and promotes apoptosis in MCL‐derived cell lines. Human MCL cell lines Jeko‐1, Mino and HBL‐2 were treated with deferoxamine mesylate salt (DFO) using previously reported concentrations.^12^ A 24‐ and 48‐hour incubation with DFO decreased MCL viability (Figure 1A, upper panel) and increased apoptosis (Figure 1A, lower panel) of MCL cells. A possible cytotoxic outcome because of a high concentration of DFO was ruled out by abrogating the effect by concomitant administration of ferric ammonium citrate (FAC). The co‐incubation of cells with DFO and FAC reversed the cytotoxic effect of DFO (Figure 1A, lower panel). Post‐incubation with FAC after the pre‐incubation with DFO also reversed the effect of iron chelation further demonstrating that the deprivation of iron is a cause of growth reduction and the effect is reversible (data not shown). Treatment with FAC alone had no effect on cell growth or apoptosis (data not shown). We then examined the effect of iron depletion on the cell lines which do not have constitutively active cyclin D1, and we used SUDHL‐6 originating from diffuse large B‐cell lymphoma and DG‐75 isolated from Burkitt's lymphoma. Their growth after DFO treatment was decreased but not fully inhibited (growth rate of MCL vs non‐MCL cell lines at 48‐hour time point was significantly different, *P* < .05; Figure 1B, left panels), suggesting that the overexpression of cyclin D1 augments the sensitivity of MCL cell lines to treatment with DFO. However, the level of apoptosis after DFO treatment was comparable between non‐MCL and MCL cell lines (Figure 1B, right panel). All MCL cell lines had detectable levels of cyclin D1 at baseline; while under the same experimental conditions, the expression of cyclin D1 in non‐MCL cell lines was not detected. In MCL cell lines, cyclin D1 protein was no longer detectable on Western blot after 24 hours of incubation with DFO and FAC post‐incubation fully restored the cyclin D1 protein level (Figure 1C, left panel). In order to determine the iron chelation effect on the cell cycle progression, we synchronized MCL and non‐MCL cells by serum starvation for 24 hours and then released the cells into the medium with 10% FBS or 10% FBS with DFO (protein levels of cyclin D1 are shown on Figure 1C, right panel). Cell cycle analysis revealed that MCL cell lines do not abrogate the cell cycle in the G1 phase under serum‐starved conditions, indicating that the overexpression of cyclin D1 promotes cell proliferation (Figure 1D). In addition, the overexpression of cyclin D1 made MCL cell lines more susceptible to treatment with DFO (percentage of G1 cells of MCL cell lines was significantly higher than in non‐MCL cell lines, Figure 1D). Despite the report^12^ suggesting that *cyclin D1* mRNA is stable after iron chelation and its expression is regulated via the proteasome, DFO treatment of MCL cell lines decreased mRNA level of *cyclin D1* (Figure 1E, left panel). We also investigated the possible compensatory effect described by Klier et al,^33^ where a specific shRNA‐mediated knock‐down of *cyclin D1* mRNA had minimal effect on cell survival because of up‐regulation of *cyclin D2* mRNA and protein expression. After treatment with DFO, we did not detect altered levels of either *cyclin D2* or *cyclin D3* mRNA (Figure 1E, middle panel). We further tested whether the DFO effect may be due to inhibition of expression of one of the iron‐dependent hypoxia‐inducible factor hydroxylases. Expression analysis of MCL cell lines treated with DFO revealed down‐regulation of *EGLN2* gene and up‐regulation of *EGLN1* (Figure 1E, right panels).

### 3.2 Regulation of cyclin D1 in MCL cell lines is not controlled by EGLN2/PHD1 and its hydroxylation target FOXO3A

**Figure 2:**
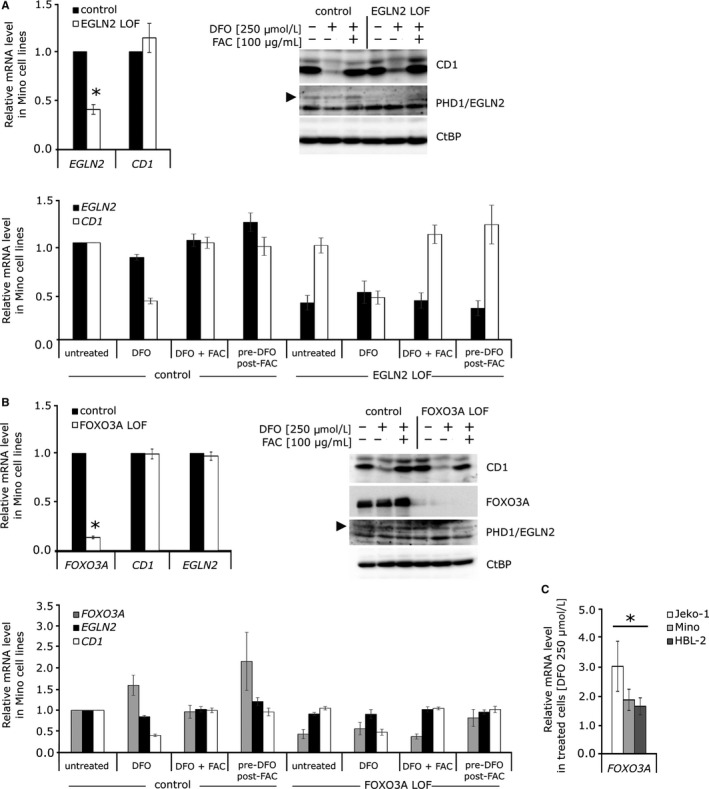
Regulation of cyclin D1 in mantle cell lymphomamantle (MCL) cell lines is not controlled by *EGLN2*/PHD1 and its hydroxylation target FOXO3A. All data are represented as the mean of three independent experiments; T bars designate standard errors. *P < .05. Expression analyses are normalized to *GUSB* and/or *RPLP0* reference gene. A. Loss of *EGLN2*/PHD1 does not affect cellular level of cyclin D1. (Upper left panel) The decreased expression of *EGLN2* after CRISPR‐Cas9 mediated loss‐of‐function in Mino cell line was determined by quantitative PCR. The expression of *cyclin D1* in these cells did not change. (Upper right panel) Parental Mino cell line and *EGLN2*/PHD1 LOF Mino cell line were treated with DFO (250 µmol/L) only or in combination with FAC (100 µg/mL) for 24 h, and protein levels of cyclin D1, PHD1 and CtBP (loading control) were determined by Western blot. (Lower panel) The expression analysis of parental Mino cell line and *EGLN2*/PHD1 LOF Mino cell line after DFO (250 µmol/L) and FAC (100 µg/mL) treatment for 24 h. B, Loss of FOXO3A does not affect cellular level of *EGLN2*/PHD1 and cyclin D1. (Upper left panel) Decreased expression of *FOXO3A* after CRISPR‐Cas9–mediated loss‐of‐function in Mino cell line was determined by quantitative PCR. The expression of *cyclin D1* and *EGLN2* in these cells did not change. (Upper right panel) Parental Mino cell line and FOXO3A LOF Mino cell line were treated with DFO (250 µmol/L) only or in combination with FAC (100 µg/mL) for 24 h, and protein levels of FOXO3A, cyclin D1, PHD1 and CtBP (loading control) were determined by Western blot. (Lower panel) The expression analysis of parental Mino cell line and FOXO3A LOF Mino cell line after DFO (250 µmol/L) and FAC (100 µg/mL) treatment for 24 h. C. FOXO3A up‐regulation after deferoxamine (250 µmol/L) treatment. Treatment with iron chelator for 24 h increased the expression of *FOXO3A* gene in MCL cell lines Jeko‐1, Mino and HBL‐2

It has been previously reported that an inability of PHD1 to hydroxylate FOXO3A promotes its accumulation in cells, which in turn suppresses cyclin D1 expression by a yet unknown mechanism.^13^ In order to decipher whether iron chelation down‐regulates cyclin D1 by inhibiting PHD1 function and thus prevents FOXO3A proteasomal degradation, we created *EGLN2* and *FOXO3A* CRISPR/Cas9 based LOF MCL Mino cell lines (Figure 2A,B). The loss of PHD1 did not lead to the down‐regulation of *cyclin D1* expression in MCL cell line (Figure 2A, upper panels). In order to validate our CRISPR/Cas9 system, we created *EGLN2* LOF in HEK293 cells, and as expected, we observed down‐regulation of *cyclin D1* on mRNA level (data not shown), suggesting that transcriptional regulation of *cyclin D1* in MCL cell lines is not controlled by PHD1. We further examined the effect of iron chelation on cyclin D1 regulation in *EGLN2*/PHD1 and FOXO3A LOF cell lines. As expected, level of cyclin D1 on protein and mRNA levels after DFO treatment was reduced and restored when iron source FAC was present in medium, in both edited cell lines (Figure 2A,2). As seen in Figure 2B, *FOXO3A* LOF in MCL line Mino did not prevent cyclin D1 down‐regulation after DFO treatment (protein level, 2B upper right panel; mRNA level, 2B lower panel). These data suggest that FOXO3A is not required for cyclin D1 repression in these cells and that cyclin D1 down‐regulation in MCL cells after DFO treatment is not directly mediated by PHD1 hydroxylase. However, DFO treatment leads to up‐regulation of *FOXO3A* transcript in parental Mino, CRISPR/Cas9 unedited cells. Up‐regulation of *FOXO3A* expression was also detected in other MCL cell lines after DFO treatment (Figure 2C).

### 3.3 Down‐regulation of EGLN2 and accumulation of FOXO3A mRNA after DFO treatment in MCL cell lines is caused by induced hypoxia

**Figure 3:**
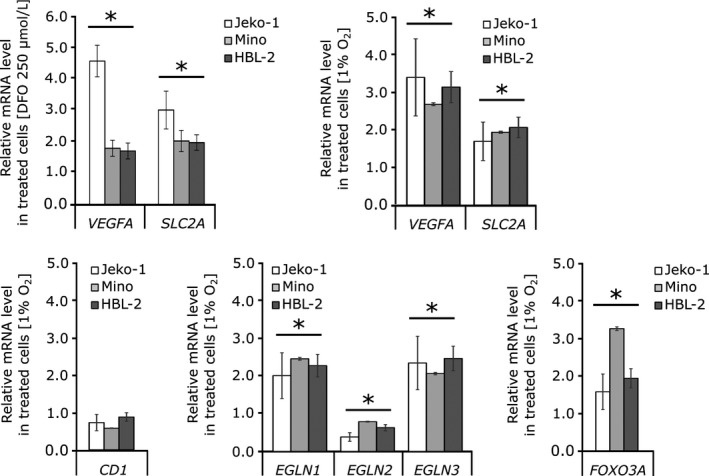
Hypoxia treatment of mantle cell lymphomamantle cell lymphoma cell lines. Expression levels of *cyclin D1*, *EGLNs*, selected HIF target genes (*VEGFA* and *SLC2A*) and *FOXO3A* were determined by quantitative PCR after incubation in hypoxia chamber with 1% O_2_ for 24 h. T bars designate standard errors. *P < .05. Expression analyses are normalized to *GUSB* and/or *RPLP0* reference gene. (Upper panels) The mRNA expression of HIF target genes (*VEGFA and SLC2A*) is significantly up‐regulated after deferoxamine and hypoxia treatment. (Lower panels) The level of *cyclin D1* was not significantly altered by hypoxia treatment, but we detected down‐regulation of *EGLN2* and accumulation of *FOXO3A* mRNA

As DFO is a known hypoxia‐mimetic agent, we asked whether the down‐regulation of cyclin D1 after DFO treatment is induced by hypoxia. First, we checked the expression of selected known HIF target genes *VEGFA* and *SLC2A*; both were up‐regulated after DFO treatment in MCL cell lines (Figure 3, upper left panel). We then cultured MCL cell lines for 24 hours at 1% O_2_ in hypoxia chamber and performed expression analyses. While hypoxia effect was confirmed by the expression of HIF target genes *VEGFA* and *SLC2A *(Figure 3, upper right panel), the level of *cyclin D1* was not significantly altered (Figure 3, lower left panel). However, we detected down‐regulation of *EGLN2* and accumulation of *FOXO3A* mRNA (Figure 3, lower panels). It has been previously shown that *EGLN2* promoter contains binding sites for aryl hydrocarbon nuclear translocator (*ARNT*/HIF‐1β)^34^ which mediates its down‐regulation under hypoxic conditions and that *FOXO3A* transcript level, in response to hypoxia, accumulates in HIF1‐dependent manner, resulting in enhanced FOXO3A activity.^35^ Here, our data demonstrate that down‐regulation of *EGLN2* and accumulation of *FOXO3A* mRNA after DFO treatment is rather a consequence of induced hypoxia created by iron depletion, but neither hypoxia nor *ENGL2*/PHD1‐FOXO3A pathway alone regulate cyclin D1 expression in MCL cells.

### 3.4 Treatment with prolyl hydroxylase inhibitor DMOG decreases MCL cells’ viability

**Figure 4:**
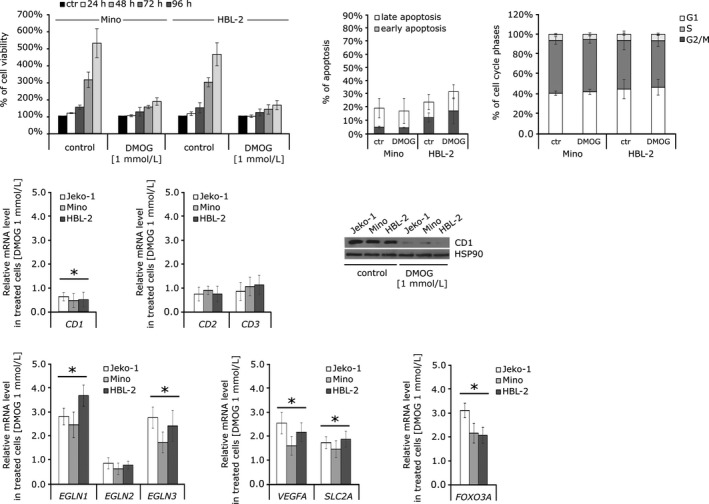
The effect of dimethyloxalylglycine (DMOG) (1 mmol/L) on mantle cell lymphoma (MCL) cell lines. (Upper left panel) Proliferation rates during DMOG treatment. Inhibition of 2‐OG‐dependent enzymes has a significant effect on growth of Mino and HBL‐2 cell line compared to a control treated with vehicle DMSO (0.2%). Cell number and viability were determined using CellTitre‐Blue reagent (Promega) and Perkin‐Elmer Envision analyzer. The results are demonstrated as percentage of cell growth in comparison with the number of cells at time 0. (Upper middle panel) Percentage of total apoptotic cells (divided into early and late fractions) during DMOG treatment, 72 h. (Upper right panel) Cell cycle distribution after DMOG treatment, 72 h. Data are demonstrated as percentage of cells in G1, S and G2/M cell cycle phases. (Middle left panels) Treatment with DMOG decreases cellular cyclin D1 level in MCL cell lines. The expression of cyclin D1 was reduced in MCL cell lines after incubation with DMOG for 24 h on protein and also mRNA level. The mRNA expression of *cyclin D1* homologs, *cyclin D2 *(*CD2*) and *cyclin D3 *(*CD3*) is not significantly affected by DMOG treatment. (Middle right panel) Treatment with DMOG depleted cyclin D1 protein level. The level of cyclin D1 was decreased in MCL cell line treated with DMOG already after 72 h. (Lower panels) Expression levels of *EGLNs*, selected HIF target genes (*VEGF* and *SLC2A*) and *FOXO3A* were determined by quantitative PCR after incubation with DMOG for 24 h

Despite the fact that direct PHD1 hydroxylase inactivation does not seem to influence regulation of cyclin D1 in MCL, we asked whether inhibition of 2‐OG‐dependent hydroxylases could impact the MCL cells. We treated MCL cell lines with prolyl hydroxylase inhibitor DMOG, a synthetic analogue of 2‐OG, which catalytically inhibits hydroxylation reaction. We found decreased proliferation rate of MCL cells (Figure 4, upper left panel), their decreased cyclin D1 protein and mRNA levels, while expression of cyclin D1 homologs, *cyclin D2* and *D3* was unchanged (Figure 4, middle, right panels). Treatment with DMOG did not result in apoptosis of MCL cells and did not affect cell cycle distribution (Figure 4, upper, middle and right panels). DMOG, similarly to DFO, mimics hypoxia and controls the expression of endogenous HIF target genes including down‐regulation of the *EGLN2* and up‐regulation of the *FOXO3A* mRNA (Figure 4, lower panels). As DMOG is predicted to inhibit a broad spectrum of dioxygenases, including hydroxylases, it is possible that these enzymes have additional substrates with the ability to regulate aberrantly expressed *cyclin D1* in MCL cells.

### 3.5 DNA damage is induced in MCL cells treated with DFO but not with DMOG

**Figure 5:**
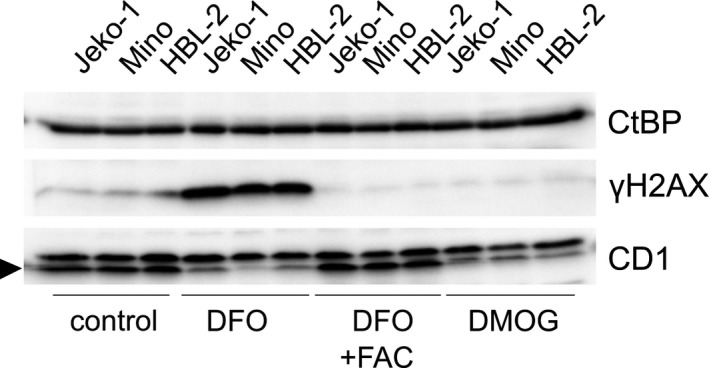
The effect of cellular iron depletion and 2‐OG‐dependent enzymes inhibition on DNA damage in mantle cell lymphoma (MCL) cell lines. Treatment with deferoxamine induces H2AX phosphorylation on S139, a DNA damage response marker indicative of DNA damage, which is reversed by concomitant administration of FAC. No increase in γH2AX signal is detected in cells treated with DMOG. CtBP was used as a loading control, and as a functional control, cellular cyclin D1 level in MCL cell lines is monitored (during the time course of the project, both batches of CD1 antibody (#2922S, Cell Signaling, lot:3) have started to detect unspecific band with higher molecular weight than cyclin D1, and cyclin D1 is indicated with an arrow)

Iron chelation inhibits multiple enzymes functioning in DNA replication, DNA repair and cell cycle progression.^8^, ^9^ One of these enzymes is RR, inhibition of which leads to dNTP deficiency.^10^ Decreased dNTP pools are known to induce DNA damage and replication stress in oncogene expressing proliferating cells.^11^, ^36^, ^37^, ^38^ We first tested whether dNTP deficiency resulting from DFO‐mediated RR inhibition causes DNA damage and apoptosis in fast proliferating cells. For this purpose, we used mouse embryonic stem cells (mESCs), suffering from intrinsic deficiency of dNTP pools, exhibiting intrinsically high phosphorylation of histone H2AX (γH2AX), a DNA damage response (DDR) marker,^39^ and displaying high sensitivity to further dNTP depletion^40^ (see Appendix S1). These experiments revealed that DFO‐mediated inhibition of RR activity causes DNA damage, DDR and apoptosis through depletion of dNTP pools, as it can be rescued by addition of deoxynucleosides to the media (Figures S1 and S2). As these processes were shown to be p53‐activation/caspase 3 cleavage dependent,^41^ we did not expect them to be induced by DMOG treatment, which is known to inhibit PHD3‐mediated hydroxylation of p53, preventing its accumulation and apoptotic activity.^18^ Indeed, when we explored the impact of DFO and DMOG treatment on DNA damage, reflected by DDR, in MCL cell lines, visible increase of γH2AX signal indicative of DDR was detected in cells treated with DFO, but not in cells treated with DMOG (Figure 5). This effect was reversed by concomitant administration of FAC with DFO. These data confirm that in addition to overlapping function of DFO and DMOG in iron‐ and 2‐OG‐dependent dioxygenase inhibition (including inhibition of PHDs), intracellular iron depletion by DFO causes DNA damage through essential function of iron in iron‐requiring enzymes involved in DNA replication, repair and cell cycle control, with predominant role of RR.^8^, ^9^

## 4 DISCUSSION

In our experiments, we tested DFO, a potent iron chelator that induces G1/S arrest and/or apoptosis in many somatic cell types, including cancer cell lines,^8^, ^12^, ^42^ as a potential non‐cytotoxic therapeutic strategy for the treatment of MCL. The causes of DFO‐mediated iron depletion–associated cell cycle perturbations and apoptosis observed in multiple cancer cell types involve mainly dNTP deficiency, resulting from inactivation of RR in DFO‐treated cell lines,^8^, ^10^ and down‐regulation of cyclin D1 because of the mechanisms that are not yet unequivocally established.^8^, ^12^, ^13^ Cyclin D1 has been postulated a therapeutic target in MCL because of its pro‐proliferation and anti‐apoptotic function in MCL^43^; therefore, iron chelation targeting cyclin D1 could represent a promising alternative treatment strategy. However, addiction of MCL cells to cyclin D1 makes it difficult to dissect a contribution of deficient dNTPs to chelators’ toxicity when cyclin D1 is down‐regulated and RR is inactivated.

To unravel the specific molecular targets that trigger cell cycle arrest and apoptosis of hyperproliferative cells exposed to DFO, we used mESCs that are characterized by intrinsic deficiency of dNTP pools and high intrinsic replication stress as a model.^39^, ^40^ Using 10 µmol/L DFO concentration (corresponding to the DFO levels achieved in patients’ plasma^44^), we showed that DFO treatment of mESCs leads to critical deficiency of nucleotides needed for DNA synthesis, suprathreshold DNA damage and apoptosis. Additionally, this effect was to a large extent rescued by deoxynucleoside supplementation to the media. Cyclin D1 down‐regulation also results in inhibition of cell cycle progression and cell cycle arrest in proliferating cancer cells; nevertheless, it was postulated that its down‐regulation by iron depletion is mediated by other than RR activity–related mechanisms,^8^, ^42^ likely involving PHDs from the iron and 2‐OG‐dependent dioxygenase family.^12^, ^13^

Therefore, we tested the effect of inhibition of the human 2‐OG‐dependent prolyl hydroxylases on MCL cells. Cells treated with DMOG had decreased proliferation and down‐regulated cyclin D1 at mRNA and protein level, but in contrast to DFO‐treated MCL cells, we were unable to detect their increase of apoptosis and their cell cycle arrest. The detection of increased DNA damage marker γH2AX indicative of DNA damage in cells treated with DFO, but not in MCL cells treated with DMOG, confirmed differences in DNA damage induction of these agents. DFO and DMOG have overlapping impact on *cyclin D1* expression (we postulate as a result of yet unknown iron and 2‐OG‐dependent dioxygenases inhibition), but DFO treatment is superior to DMOG in inhibition of proliferation of MCL cells because of its ability to suppress iron‐dependent RR.^11^

As the role of iron in regulation of *cyclin D1* expression is not completely understood, we investigated the molecular mechanism underlying decreased cyclin D1 mRNA and protein levels in MCL cell lines after DFO‐induced iron deficiency. PHDs are dependent on iron^45^ to catalyse its hydroxylation activity; thus, iron chelation decreases their enzyme activity. Nevertheless, *EGLN2*/PHD1 LOF in Mino cells did not affect *cyclin D1* expression and *FOXO3A* LOF did not restore cyclin D1 levels after chelation treatment. Therefore, the cyclin D1 in MCL cells escapes this regulation circuit and its down‐regulation by iron depletion is mediated by another, yet unknown mechanism(s). We hypothesize that in MCL cells production of cyclin D1, which is aberrantly localized in the proximity of a nucleolus and influenced by specific transcription enhancers (eg, nucleolin)^46^ because of t(11;14) translocation, escapes the PHD1‐FOXO3A regulation. It is known that iron chelators enhance HIFs‐α accumulation^47^ and thus induce hypoxia response. We measured expression of *cyclin D1*, *EGLN2* and known HIF target genes *VEGF* and *SLC2A* after 24 hours in 1% O_2_ hypoxia and found that *cyclin D1* level was not altered, but *EGLN2* expression was down‐regulated, suggesting that its down‐regulation after DFO treatment is caused by hypoxia. FOXO3A is a transcription factor known to be involved in many cellular processes such as apoptosis,^48^, ^49^, ^50^ autophagy,^51^ oxidative stress^51^ and DNA repair.^52^ We ruled out the role of FOXO3A in cyclin D1 repression because of iron depletion, but we observed accumulation of *FOXO3A* after chelation treatment as a result of induced hypoxia. In many MCL tumour tissues, FOXO3A is constitutively inactivated and it was reported that its reactivation by nuclear export inhibitors had profound impact on cell viability.^53^ We can only speculate that *FOXO3A* induction by chelation treatment would be also beneficial for MCL therapy.

In conclusion, iron chelation and treatment with non‐selective hydroxylase inhibitor DMOG,^54^ or by other 2‐OG‐dependent dioxygenase inhibitors (eg, FG4497, data not shown), decrease MCL cell lines proliferation by down‐regulating cyclin D1 mRNA and protein levels. These data support further exploration of the use of iron chelation and 2‐OG‐dependent enzyme inhibitors as a novel therapy of MCL. Unlike in other cancer cells, the expression of *cyclin D1* in MCL is neither regulated by *EGLN2*/PHD1 nor by FOXO3A; thus, the molecular mechanism controlling cyclin D1 production and degradation in MCL remains to be elucidated.

## CONFLICT OF INTEREST

The authors declare no competing financial interest.

## AUTHOR'S CONTRIBUTIONS

O. Babosova performed the research and drafted the manuscript. K. Kapralova, L. Raskova Kafkova and L. Lanikova performed the research and analysed the data. V. Korinek and JT Prchal participated on study design/co‐ordination and critically revised the manuscript. V. Divoky and L. Lanikova designed the research and drafted the manuscript. All authors have read and approved the final article.

## Data Availability

The data that support the findings of this study are available from the corresponding author upon request.
