# Supplementary material for: Iron chelation and 2‐oxoglutarate‐dependent dioxygenase inhibition suppress mantle cell lymphoma's cyclin D1
Source: J Cell Mol Med. 2019 Sep 13;23(11):7785–95. doi: 10.1111/jcmm.14655 (PMC6815829; doi:10.1111/jcmm.14655)
Supplement: Supplementary file 1 [file JCMM-23-7785-s001.pdf]

Supplementary Figures

FIGURE 1.

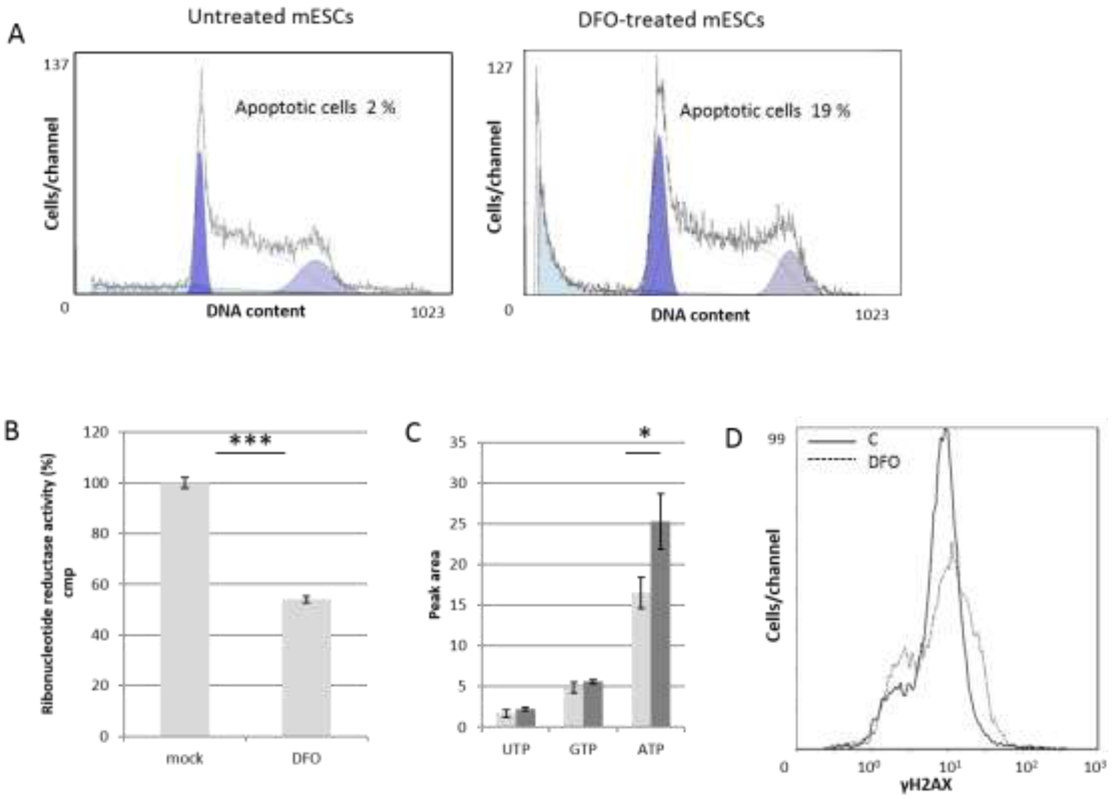

A. Cell cycle analysis of untreated and DFO-treated mouse embryonic stem cells (mESCs). The number of apoptotic cells was determined as a sub-G1 peak. DFO significantly increases the number of apoptotic cells.

B. Ribonucleotide reductase (RR) activity in untreated and DFO-treated mESCs was determined after pulse of cells with [14C]-cytidine (1.25 µCi) for 30 min at 37°C as the amount of radioactivity emitted by genomic DNA. The data shown are means ± SD from three independent experiments. The results were normalized to mock-treated cells. Statistical significance was assessed using Student's *t*-test, $^{***}P < 0.001$. DFO significantly decreases RR activity to 57% of its original activity.

C. Analysis of ribonucleotides in untreated (light bars) and DFO-treated mESCs (dark bars) by capillary electrophoresis. The data shown are means ± SD from three independent experiments. Statistical significance was assessed using Student's *t*-test, $^{*}P < 0.1$. DFO leads to cellular accumulation of ribonucleotides.

D. Untreated (C) and DFO-treated (DFO) mESCs were stained with FITC-labeled anti-γH2AX antibody and fluorescence was measured by flow cytometry and determined as a mean fluorescence intensity (MFI).

The MFI values are as follows: for untreated cells: 8.3; for DFO-treated cells: 9.5, the figure shows representative experiment of 3 independent experiments. DFO leads to accumulation of γH2AX.

FIGURE 2.

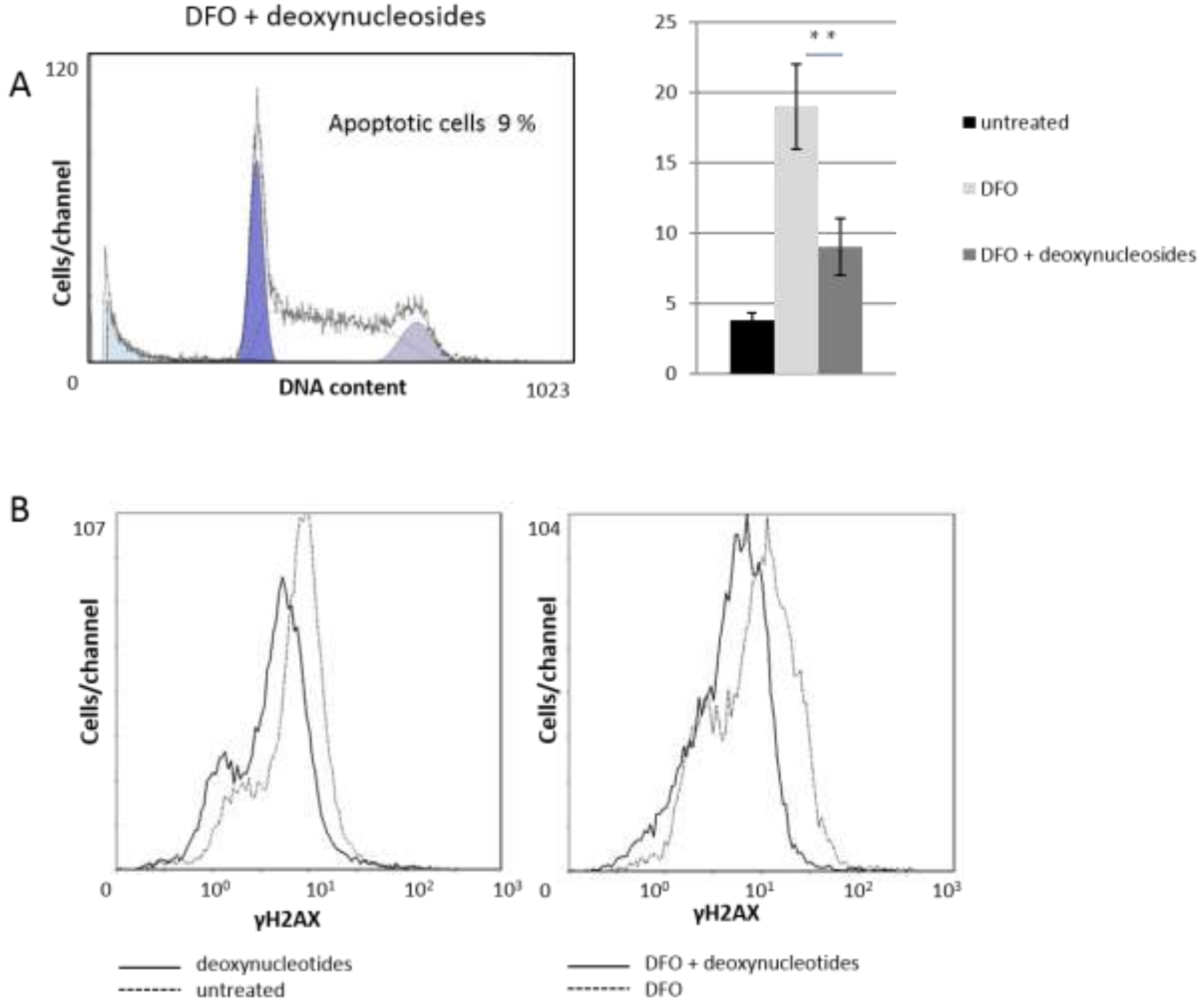

A. Cell cycle/apoptosis analysis of deoxynucleoside-supplemented and DFO-treated mESCs. The co-presence of deoxynucleosides and DFO in cultivation medium leads to significant decrease of apoptotic cells in comparison with DFO treatment only. Statistical analysis of the percentage of apoptotic cells, shown in the graph on right, was assessed using Student's *t*-test, $^{**}P < 0.01$.

B. Supplementation of mESC culture media with deoxynucleosides leads to decrease of intrinsically expressed γH2AX. Correspondingly, addition of deoxynucleosides decrease γH2AX in mESCs treated with DFO. The MFI values are as follows: for untreated cells: 8.3; for DFO-treated cells: 9.9; for cells treated with deoxynucleosides: 6.5 and for DFO-treated and nucleoside-supplemented cells: 6.3.
