# Supplementary material for: Iron chelation and 2‐oxoglutarate‐dependent dioxygenase inhibition suppress mantle cell lymphoma's cyclin D1
Source: J Cell Mol Med. 2019 Sep 13;23(11):7785–95. doi: 10.1111/jcmm.14655 (PMC6815829; doi:10.1111/jcmm.14655)
Supplement: Supplementary file 2 [file JCMM-23-7785-s002.pdf]

Supplementary Materials and Methods

Cell culture

F1 hybrid 129SvJae×C57BL/6 mouse ESC (mESC) line V6.5 (Open Biosystems) was maintained on culture dishes covered with 0.1% gelatin (Sigma-Aldrich) in a humidified atmosphere of 10% CO_2 at 37°C in high-glucose Dulbecco's modified Eagle's medium (D-MEM) with GlutaMAX (Gibco) supplemented with 15% fetal bovine serum (FBS; Hyclone), 0.1 mM non-essential amino acids, 1 mM sodium pyruvate, 2 mM glutamine, 100 U/mL penicillin, 100 μg/mL streptomycin (Gibco), 0.1 μM β-mercaptoethanol (Serva) and 1000 U/mL leukemia inhibitory factor (ESGRO, Chemicon). The total *in vitro* culture time of these mESCs at the time of experimental testing was equivalent to passage number 22-40 of the original mESC line. 10 μM deferoxamine mesylate (DFO, Sigma-Aldrich) was used when appropriate. The cells were checked repeatedly for chromosome number and possible karyotypic abnormalities. The rescue experiment was performed using mixture of deoxynucleosides namely 2′-deoxyadenosine, 2′-deoxyguanosine, 2′-deoxycytidine, 2′-deoxythymidine (purchased individually from Sigma-Aldrich), concentration was 50 μM each. Deoxynucleosides were added at the same time as DFO when appropriate or used separately.

Determination of RR activity

Exponentially growing cells ($5x10^5$) were treated with 10 μM DFO or left untreated. Then, cells were pulsed with ^{14}C-cytidine (100 μCi/mL, Moravek Biochemical, CA) at 37°C for 30 min, collected, washed twice with PBS and processed to extract total genomic DNA. Thereafter, high-efficiency liquid-scintillation counting of ^{14}C-labelled DNA was used. ^{14}C-cytidine was quantified simultaneously using an LS6500 liquid scintillation counter (Beckman Coulter).

Determination of intracellular nucleotides

To identify intracellular nucleotides, DFO-treated cells and untreated cells ($5×10^6$ cells of each) were extracted in 100 μl of ice-cold 0.1 M TCA. Samples were left on ice (10 min) for deproteination; then, after centrifugation (3 min, 12 100×g), the supernatant was collected and back-extracted with diethyl ether (3×1 mL). The water phase was analyzed by capillary

electrophoresis on an Agilent 3D CE instrument (Agilent Technologies, Waldbronn, Germany) as described in Friedecky et al., 2007.

Flow cytometric analysis

Cells were collected in time points as indicated, washed in PBS+1% FBS, fixed with 70% ethanol, and stained with 60 µg/µl propidium iodide (Sigma-Aldrich) in 1.1% sodium citrate dehydrate with 5 ng/µl ribonuclease A (DNA Lego Ribonuclease A; Top-Bio, Czech Republic) for 30 min at 37°C in the dark, and analyzed by flow cytometry. For γH2AX analysis, the cells were fixed in 70% ethanol, and stained with anti Phospho-Histone H2A.X (Ser139) (20E3) Rabbit mAb (Alexa Fluor® 488 Conjugate, 1:50. Cell Signaling Technology) for 45 min at 37°C in the dark and analyzed by flow cytometry. All flow cytometry analyses were performed on the Cytomics FC 500 machine using a CXP software (Beckman Coulter) by standard procedure.

Statistical analysis

Presented data were described as absolute numbers and percent prevalence; continuous variables are presented as average ± standard deviation. Student's t-test was used to compare continuous variables. A p-value of less that 0.05 was considered statistically significant.

Reference

Friedecký D, Tomková J, Maier V, *et al*. Capillary electrophoretic method for nucleotide analysis in cells: Application on inherited metabolic disorders. *Electrophoresis*. 2007; 28: 373-380.
